# Molecular mapping across three populations reveals a QTL hotspot region on chromosome 3 for secondary traits associated with drought tolerance in tropical maize

**DOI:** 10.1007/s11032-014-0068-5

**Published:** 2014-03-16

**Authors:** Gustavo Dias Almeida, Sudha Nair, Aluízio Borém, Jill Cairns, Samuel Trachsel, Jean-Marcel Ribaut, Marianne Bänziger, Boddupalli M. Prasanna, Jose Crossa, Raman Babu

**Affiliations:** 1 Universidade Federal de Viçosa (UFV), CEP: 36.570-000 Viçosa, Minas Gerais State Brazil; 2 International Maize and Wheat Improvement Center (CIMMYT), Apdo. Postal 6-641, Mexico, DF Mexico; 3 CIMMYT, ICRAF House, United Nations Avenue, Gigiri, Nairobi, 00621 Kenya; 4 Generation Challenge Program, Hosted by CIMMYT, Apdo. Postal 6-641, Mexico, DF Mexico; 5 Monsanto Company, CEP: 38.405-232 Uberlândia, Minas Gerais Brazil

**Keywords:** Secondary traits, MetaQTL, Drought tolerance, SNP

## Abstract

Identifying quantitative trait loci (QTL) of sizeable effects that are expressed in diverse genetic backgrounds across contrasting water regimes particularly for secondary traits can significantly complement the conventional drought tolerance breeding efforts. We evaluated three tropical maize biparental populations under water-stressed and well-watered regimes for drought-related morpho-physiological traits, such as anthesis-silking interval (ASI), ears per plant (EPP), stay-green (SG) and plant-to-ear height ratio (PEH). In general, drought stress reduced the genetic variance of grain yield (GY), while that of morpho-physiological traits remained stable or even increased under drought conditions. We detected consistent genomic regions across different genetic backgrounds that could be target regions for marker-assisted introgression for drought tolerance in maize. A total of 203 QTL for ASI, EPP, SG and PEH were identified under both the water regimes. Meta-QTL analysis across the three populations identified six constitutive genomic regions with a minimum of two overlapping traits. Clusters of QTL were observed on chromosomes 1.06, 3.06, 4.09, 5.05, 7.03 and 10.04/06. Interestingly, a ~8-Mb region delimited in 3.06 harboured QTL for most of the morpho-physiological traits considered in the current study. This region contained two important candidate genes viz., *zmm16* (MADS-domain transcription factor) and *psbs1* (photosystem II unit) that are responsible for reproductive organ development and photosynthate accumulation, respectively. The genomic regions identified in this study partially explained the association of secondary traits with GY. Flanking single nucleotide polymorphism markers reported herein may be useful in marker-assisted introgression of drought tolerance in tropical maize.

**Electronic supplementary material:**

The online version of this article (doi:10.1007/s11032-014-0068-5) contains supplementary material, which is available to authorized users.

## Introduction

Maize (*Zea mays* L.) is an important economic crop and, due its high yield potential, is currently recognised as a major crop that can ensure food security worldwide. Water scarcity is the most important environmental limiting factor for maize productivity in tropical and subtropical regions (Messmer et al. 2011). It has been projected that by the year 2050, a 70 % increase in global food production must occur, while the global climate change scenario tends to increase the problems of food insecurity (Varshney et al. 2010). This grim forecast has forced plant scientists to breed cultivars that can be grown in marginal areas with limited water availability. The genetic improvement for water stress tolerance can ensure sustainable and long-term benefits, especially when combined with improved agronomic techniques (Duvick 2005). Drought stress can adversely affect many aspects of maize physiological metabolism and growth, including photosynthesis, plant height, dry matter production, leaf area and grain yield (Ge et al. 2012). Plants undergo various morphological, biochemical and physiological changes to respond and adapt in order to survive under drought stress (Lu et al. 2011).

Increasing grain yield (GY) is the primary objective of breeding for drought tolerance; however, direct selection for GY under water scarcity has generally led to limited progress and stability owing to the reduction in the genotypic variance of GY under drought stress conditions. Secondary/morpho-physiological traits that are correlated with drought tolerance can experience increased genetic variance and heritability under stress conditions (Tuberosa et al. 2002). It has been demonstrated that some secondary traits, such as anthesis-silking interval (ASI), ears per plant (EPP), plant height (PH) and stay-green (SG) traits (leaf senescence and chlorophyll contents), are correlated with drought responses and remain stable under drought stress or might even exhibit enhanced genetic variance (Bolaños and Edmeads 1996; Betrán et al. 2003; Messmer et al. 2009; Lu et al. 2011; Messmer et al. 2011). Thus, these traits are considered useful to improve selection efficiency for drought tolerance and accordingly their use has been suggested for the improved tolerance of maize to drought and low nitrogen conditions (Bänzinger and Lafitte 1997; Bänzinger et al. 2000; Betrán et al. 2003; Lu et al. 2011). Ideally, a desirable secondary trait should be genetically correlated with GY, exhibit adequate genetic variability, record moderate to high heritability, easy and economical to measure in the field, lend itself for reliable assessments with individual plants or in small plots and have no association with poor GY in unstressed/optimal environments (Monneveux et al. 2008; Ribaut et al. 2009; Lu et al. 2011).

The value of secondary traits for drought tolerance has been well demonstrated through examining genetic correlations with GY or estimating the correlated response after indirect selection for GY (Bänzinger et al. 2000; Betrán et al. 2003, Lu et al. 2011). Bolaños and Edmeads (1996) reported evaluation of 3,509 inbred lines for 50 traits under well-watered (WW) and water-stressed (WS) conditions in Mexico and detected a strong genotypic correlation at a magnitude of −0.60 and 0.90 for ASI and EPP, respectively, with GY. Chapman and Edmeades (1999) detected a genotypic correlation of −0.89, 0.95 and 0.70 between GY and ASI, EPP and the visual leaf senescence score, respectively, under drought conditions. More recently, Lu et al. (2011) reported the genetic correlation of many secondary traits under WW and WS conditions, evaluating a set of 550 lines of an association mapping panel comprising of lines from tropical, subtropical and temperate origin from CIMMYT (International Maize and Wheat Improvement Center) and CAAS (Chinese Academy of Agricultural Sciences). This study reported positive and significant association between GY and plant height, chlorophyll content on ear and leaf senescence under drought and optimal conditions. Logically, the genetic gain for GY could be higher with the use of secondary traits in a combined selection index than in the case of selection for grain yield alone (Ziyomo and Bernardo 2012). The selection efficiency in 19 maize populations under low nitrogen conditions was improved by 14 % when secondary traits, such as ASI, EPP and SG were included in the selection index over selection for grain yield alone (Bänzinger and Lafitte 1997).

Maize is more susceptible than other rain-fed cereal crops because of its near-synchronous development of florets, usually on a single ear, and the physical separation of male and female flowers on the same plant. Typically, during drought stress, the time interval between male and female flowering increases which usually results in poorer partitioning of the photosynthates to the ear (Araus et al. 2011). Leaf senescence is a type of cell death programme that is inappropriately activated in response to the degradation of chlorophyll in plants under drought conditions. Delayed leaf senescence and higher chlorophyll concentrations are associated with the stay-green capacity of plants and play an important role in enhancing drought tolerance (Rivero et al. 2007). Stay-green genotypes are associated with the retention of chlorophyll in the leaves and maintenance of the ability to undergo photosynthesis for longer periods than senescent genotypes under terminal drought conditions (Harris et al. 2007). Stay-green could be evaluated at the leaf level using portable chlorophyll metres, such as the Minolta SPAD (Cai et al. 2012a, b). Secondary traits that are easy and inexpensive to measure have been adopted in the breeding programmes (Ribaut et al. 2009). However, QTL information pertaining to such key secondary traits that are associated with drought tolerance in maize is scarce (Messmer et al. 2011). Root traits play an important role in plant adaptation to drought-prone conditions. However, practical aspects of selecting for root traits in maize is labour, resource and time intensive (Hund et al. 2011), especially if large number of genotypes are to be evaluated (Landi et al. 2010). Non-destructive measurement of root capacitance using a portable capacitance metre offers a feasible way of approximating the relative differences in the extension of the root system (Rajkai et al. 2005). However, the efficiency of root capacitance as an indicator of drought tolerance in maize breeding has not been well established till date (Lu et al. 2011; Messmer et al. 2011).

An association between morpho-physiological traits and grain yield and their use in conventional breeding programmes has been frequently demonstrated (Bolaños and Edmeads 1996; Bänzinger and Lafitte 1997; Betrán et al. 2003; Monneveux et al. 2008; Zheng et al. 2009; Lu et al. 2011). However, genetic basis of such traits, their genomic location and cause–effect relationship among various secondary traits have not yet been elucidated. Molecular marker approaches offer an important tool to understand the relationship between grain yield and secondary traits and dissect their genetic basis. The co-localisation of grain yield QTL with that of secondary traits could be an excellent indication of strong association. Identification of such QTL of secondary traits that improve crop growth and performance especially under water-limited conditions will certainly assist the breeders in rapid introgression of these genomic regions into desired elite germplasm (Landi et al. 2010; Collins et al. 2008; Swamy et al. 2011).

We evaluated three tropical biparental populations and reported QTL for GY and ASI under different water regimes (Almeida et al. 2013). Here, we present the results of QTL mapping of a number of secondary traits under managed drought and optimal conditions in the three biparental populations using single nucleotide polymorphism (SNP) and simple sequence repeats (SSR) markers. Specifically, the objectives of the present investigation were to (1) determine the heritability and relationship of secondary traits with GY under WS and WW environments; (2) identify genomic regions influencing the secondary traits across water regimes; and (3) detect QTL hotspot genomic regions, if any for secondary traits that express more or less uniformly across different genetic backgrounds to facilitate marker-assisted introgression of drought tolerance in tropical maize.

## Materials and methods

### Plant materials

Three biparental maize populations from Global Maize Program of CIMMYT were evaluated under WW and WS conditions. *Population 1* comprised of 234 recombinant inbred lines (RILs) from the cross, CML444 × MALAWI, developed using the single seed descent method. *Population 2* comprised of 247 F_2:3_ families from the cross, CML440 × CML504, obtained from randomly chosen F_2_ plants. *Population 3* comprised of 300 F_2:3_ families, obtained from randomly chosen F_2_ plants from the cross CML444 × CML441. The parental lines—CML444, CML441, CML440 and CML504 were adapted to tropical and subtropical African mid-altitude environments and considered to be tolerant to drought and low nitrogen levels. These lines have a compact phenotype with strong, erectophile and dark green leaves. SC-MALAWI is also a subtropical line with moderate tolerance to water-limited conditions, but exhibit long, horizontal and light green leaves. This inbred line was developed in southern Zimbabwe in the 1960s and has been widely used in developing hybrids in both the public and private sector especially for mid-altitude subtropical environments. Segregating RILs/families of CML444 × MALAWI and CML444 × CML441 were test crossed to CML312, whereas CML440 × CML504 was test crossed to CML395 for phenotypic evaluations.

### Field experiments

The field experiments were conducted in Mexico (Tlaltizapán station: 18ºN, 99ºW, 940 m). Phenotypic evaluation of test cross hybrids, under WW conditions, were carried out during the rainy season in 2010, and two field experiments were conducted under WS conditions during the dry season in 2010 and 2011 for each of the population. Climatologic conditions of this environment for drought phenotyping have been previously described (Masuka et al. 2012; Almeida et al. 2013). The experimental design was an alpha (0,1) lattice (Patterson and Williams 1976) with two replications and one-row plot size of 5 m, with 0.75 m between the rows. Plots were planted with two seeds per hill and thinned to one plant per hill 3 weeks after planting, resulting in a plant population of approximately 66,667 plants ha^−1^. Drought stress was applied during the flowering time in accordance with the established protocols in CIMMYT (Bänzinger et al. 2000). For WS conditions, the furrow irrigation method at 10-day intervals was used until 3 weeks before the expected anthesis date (AD) in each population. This stress condition was maintained until 5 weeks after 50 % of the families had flowered. An additional irrigation was applied during grain filling. In WW trials at all the locations, the soil moisture was maintained at field capacity.

The traits evaluated in this study were according to CIMMYT’s established protocols (Bänzinger et al. 2000; Betrán et al. 2003; Araus et al. 2011; Lu et al. 2011). A total of nine traits were measured under both the water regimes. The trait names and brief measurement descriptions are listed in Table 1. Traits with more detailed measurements are described below. Senescence and relative chlorophyll contents were measured three times repetitively at an interval of 2 weeks, after 50 % of the families had flowered. The three measurements were used to estimate the areas under curve of progress of senescence and chlorophyll contents. Root capacitance were measured in five plants per plot using a BK Precision 810A Meter (Maxtec Inc., Chicago, USA); the negative electrode was connected to the stem above the first node, and the positive electrode was connected to a rod inserted into the soil in the middle section of the furrow next to the plot under consideration (Messmer et al. 2011; Lu et al. 2011).

**Table 1:** Description of the measured traits for drought tolerance

Traits	Description
GY	Grain yield in t/ha
ASI	Anthesis-silking interval, measured as the difference between male (AD) and female flowering (SD) time, the interval time from sowing to 50 % individuals flowering in each plot
EPP	Number of ears per plant, measured as number of harvested ears with kernels by the number of plants per plot
SENES	Leaf senescence, scored using a scale from 0 to 10 (1 = 10 %; 2 = 20 %; 3 = 30 %; 4 = 40 %; 5 = 50; 6 = 60 %; 7 = 70 %; 8 = 80 %; 9 = 90 %; and 10 = 100 % dead leaf area scored at 3, 5 and 7 weeks after 50 % of the plant reached anthesis)
CEL	Chlorophyll content in the ear leaves, measured in five plants per plot at 3, 5 and 7 weeks after 50 % of the plants reached at anthesis using a SPAD metre
CYL	Chlorophyll content in young leaf (second leaf from tassel) measured in five plants per plot at 3, 5 and 7 weeks after 50 % of the plants reached anthesis using a SPAD metre
PH	The average height from ground to the tassel tip in five plants scored randomly in each plot
EH	The average height from ground to the node bearing the highest ear in five plants scored randomly in each plot
PEH	Plant-to-ear height ratio
RC	Root capacitance, measured using an electrical capacitance metre at 2 days after a grain filling irrigation

Abbreviation of traits names

### Phenotypic data analysis

The raw plot data were analysed in linear mixed model in PROC Mixed of SAS using REML. In WS conditions, the two field experiments per population were pooled using a combined analysis. The adjusted means for each line were estimated using the following linear model:$Y_{ijk} = \mu + \text{Re} + B_{j} (\text{Re} ) + G_{k} + \varepsilon_{ijk}$, where *Y* is the trait of interest, *μ* is the mean effect, Re is the effect of the *i*th replicate, *B*_*j*_(Re) is the effect of the *j*th incomplete block within the *i*th replicate and *G*_*k*_ is the effect of the *k*th genotype. In the case of combined data from 2 year terms, *E*_*i*_ and (*E*_*i*_ × *G*_*l*_) were incorporated into the linear model, where, *E*_*i*_ is the effects of the *i*th environment and *E*_*i*_ × *G*_*l*_ represents the environment × genotype interaction. To estimate the Best Linear Estimated Value (BLUEs), the genotypes were considered as fixed terms, while all other terms were declared random. The broad-sense heritability (*H* ^2^) was estimated using the formula: $H^{2} = {{\sigma_{\text{g}}^{2} } \mathord{\left/ {\vphantom {{\sigma_{\text{g}}^{2} } {\left( {\sigma_{\text{G}}^{2} + \sigma_{\text{GE}}^{2} /l + \sigma^{2} /lr} \right)}}} \right. \kern-0pt} {\left( {\sigma_{\text{G}}^{2} + \sigma_{\text{GE}}^{2} /l + \sigma^{2} /lr} \right)}}$, where $\sigma_{\text{G}}^{2}$ is the genotypic variance, $\sigma_{\text{GE}}^{2}$ is the genotype × environment interaction, *σ* ^2^ is the error variance, (*l*) is the number of environments and (*r*) is the number of replications in each trials. The genetic correlations among traits corresponded to the ratio between the genotypic covariance for each pair of traits and the product of the respective genotypic standard deviation. The phenotypic correlations among traits were calculated as simple Pearson’s correlation coefficients based on adjusted and standardised phenotypic data.

### QTL identification

**Fig. 1:**
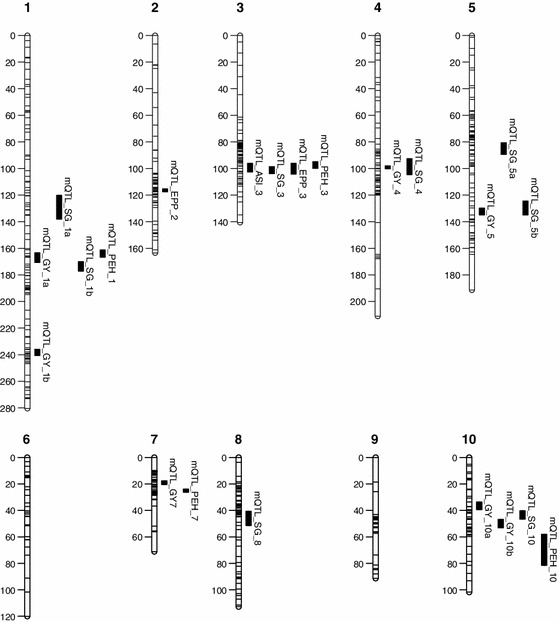
Meta-QTL for grain yield (GY), anthesis-silking interval (ASI), ears per plant (EPP), stay-green (SG) and plant-to-ear height ratio (PEH) traits represented in the consensus map of the three maize tropical populations viz., CML444 × MALAWI, CML440 × CML504 and CML444 × CML441

Individual linkage maps for each population were constructed using QTL IciMapping ver. 3.2, as described by Almeida et al. (2013). A brief description of the linkage maps in each population is given below. For the RILs of CML444 × MALAWI, a linkage map of 2,349.23 cM was constructed using the allelic information from 216 SNPs and 160 SSR markers. In the F_2:3_ populations of CML440 × CML504 and CML444 × CML441, linkage maps were constructed using 194 and 265 SNPs covering a total of 2,712.30 and 3,558.33 cM of the maize genome, respectively. To identify common genomic regions across the populations, three distinct genetic maps were merged into an integrated map using MetaQTL software version 1.0 (Veyrieras et al. 2007). The distances between the adjacent markers from all individual maps were rescaled in Haldane units. After integrating the three maps, a consensus map of 620 markers was obtained. The consensus map had a total length of 1,484.45 cM with an average distance of 2.39 cM between the markers (Fig. 1).

Individual QTL mapping analyses were performed for EPP, SENES, CEL, CYL, EH and PH in both the water regimes. QTL mapping for GY and ASI in these three biparental populations were previously performed (Almeida et al. 2013). Root capacitance was not included in the QTL mapping study due to lack of association with GY under WS conditions. The QTL were identified for the adjusted means for each trait using inclusive composite interval mapping (ICIM) (Li et al. 2007) implemented in the integrated software, QTL IciMapping v.3.2 (http://www.isbreeding.net). In all populations, the walking step in QTL scanning was 1 cM, and a likelihood odds (LOD) threshold of 2.5 was chosen for declaring potentially significant QTL for secondary traits associated with drought tolerance (Ribaut et al. 1997; Tuberosa et al. 2002). For F_2:3_ populations, the additive (A) and dominance (D) effects for each QTL were used to calculate the ratio of the dominance level |D/A| and classified according to Stuber et al. (1987). The QTL were considered as additive (A) = 0 − 0.20; partially dominant (PD) = 0.21–0.80; dominant (D) = 0.81–1.20; and overdominant (OD) > 1.20. The sign of the additive effects of each QTL was used to identify the origin of the favourable alleles in accordance with Lubbersted et al. (1997).

Meta-QTL analysis was performed using the QTL identified by individual QTL analyses in each of the population and environment with the MetaQTL software version 1.0 (Veyrieras et al. 2007). The well-correlated traits were merged into a single trait in meta-analysis in the trait ontology tree. Senescence and chlorophyll contents in the ear and youngest leaves were described as an integrated trait, designated ‘stay-green’ (SG), PH and EH were declared as the plant/ear height ratio (PEH), and EPP was considered a standalone trait. Meta-QTL for GY and ASI were obtained from previous results (Almeida et al. 2013). Only regions that harboured at least one QTL from each of the three populations or a higher number of QTL from a minimum of two populations were declared as meta-QTL. Detailed explanations concerning the meta-QTL analysis were previously described (Danan et al. 2011).

## Results

### Trait variation, heritability and correlation estimates under contrasting water regimes

The estimated means and heritability for each trait in all the three populations are listed in Table 2. We observed from WW to WS conditions, a marked reduction in yield in the RILs and F_2:3_ families. The yield reductions in the populations of CML444 × MALAWI, CML440 × CML504 and CML444 × CML441 were approximately 46, 40 and 49 %, respectively. The heritability estimates for GY under WS $\left( {h_{\text{GYws}}^{2} } \right)$ ranged from 0.22 to 0.54, while under WW $\left( {h_{\text{GYww}}^{2} } \right)$, ranged from 0.46 to 0.72. The heritability estimates for EPP were more or less similar in both the water regimes and $h_{\text{EPPww}}^{2}$ ranged from 0.0 to 0.34 and $h_{\text{EPPws}}^{2}$ from 0.09 to 0.31. The null $h_{\text{EPPww}}^{2}$ values in the population CML444 × MALAWI was due to the reduced variability in this trait; almost all genotypes from this population contained one ear per plant. A major reduction in EPP (13 %) from WW to WS was detected in the population, CML444 × MALAWI, followed by 7 and 5 % for CML440 × CML504 and CML444 × CML441, respectively. Smaller reductions in the latter two populations were not surprising as both the parental lines of each cross were drought tolerant, while in the RIL families, MALAWI was drought sensitive.

**Table 2:** Mean and heritability (*h* ^2^) estimates for traits tested under WW and WS conditions among the three biparental populations

Trait	Treat.	CML444 × MALAWI			CML440 × CML504			CML444 × CML441		
		Mean	*h* ^2^	*CV*	Mean	*h* ^2^	*CV*	Mean	*h* ^2^	*CV*
GY	WW	9.57	0.46	18.0	8.61	0.73	11.3	10.81	0.72	10.7
(t/ha)	WS	5.11	0.22	29.8	5.19	0.28	16.1	5.52	0.54	17.1
ASI	WW	0.74	0.39	NA	0.84	0.25	103.2	0.34	0.47	185.5
(days)	WS	3.16	0.30	53.4	3.53	0.38	33.8	3.89	0.63	26.4
PH	WW	246.94	0.63	4.3	232.36	0.62	4.4	253.37	0.5	4.1
(cm)	WS	223.7	0.40	4.6	211.86	0.52	3.6	215.75	0.53	3.3
EH	WW	139.95	0.79	4.0	138.28	0.45	7.1	139.37	0.73	4.9
(cm)	WS	128.35	0.52	6.8	118.24	0.56	5.2	115.61	0.72	4.8
EPP	WW	0.99	0.00	9.4	0.97	0.26	6.1	1.00	0.34	6.0
(uni)	WS	0.86	0.09	12.9	0.92	0.22	7.5	0.92	0.31	6.7
SENES	WW	3.63	0.07	17.1	4.69	0.20	15.1	6.64	0.28	15.3
(uni)	WS	7.63	0.27	12.3	8.21	0.27	8.9	7.65	0.44	11.1
CEL	WW	12.77	0.05	10.5	12.4	0.26	12.4	14.61	0.27	13.6
(uni)	WS	11.64	0.37	6.8	11.09	0.30	10.9	12.82	0.4	8.1
CYL	WW	9.78	0.07	12.9	8.52	0.09	10.7	10.87	0.18	10.2
(uni)	WS	7.87	0.16	11.4	6.47	0.00	5.1	8.75	0.51	5.5
RC	WW	3.99	0.33	28.4	2.25	0.00	36.8	4.36	0.47	24.4
(200nF)	WS	6.67	0.35	19.1	7.24	0.22	17.2	6.73	0.26	19.8

SENES, CEL and CYL are expressed as the area under the curve based on three measurements divided by a constant (*k* = 100)

In general, the interval between male and female flowering (ASI) was significantly high under WS conditions. Across the three populations, the average ASI under WW was 0.64 days, while under WS, the average ASI was 3.5 days, indicating an increase of greater than 80 %. Under WS conditions, the genetic variance of ASI increased substantially. The heritability estimates for ASI under WW ranged from 0.25 to 0.47, while under WS, the values ranged from 0.30 to 0.63. Similar patterns of higher heritability estimates under WS than WW conditions for the SG group of traits—leaf senescence (SENS), chlorophyll content in ear leaf (CEL) and chlorophyll content in young leaf (CYL) were observed. While drought stress in general reduced the genetic variance for GY, it did increase the variance for ASI and SG traits. In addition, the magnitude of heritability estimates for the secondary traits such as ASI, SENES and CEL were higher than that of GY under WS conditions, which indicated possible advantages of incorporating these traits into the selection index for greater genetic gains under WS conditions.

The phenotypic (*r*_p_) and genotypic (*r*_g_) correlations between GY and the morpho-physiological traits are listed in Tables 3 and 4, respectively. For certain traits, it was not possible to estimate genotypic correlation values because of the low genetic variance of the trait, which was identified as NA^ф^ (Table 4). The ASI was negatively correlated with GY. Under WS, the correlation was significant and high, whereas these two traits were weakly or not correlated under WW conditions. This was consistent with the previous reports (Bolaños and Edmeads 1996; Betrán et al. 2003), which identified ASI as an important drought adaptive mechanism in maize. The increase in the ASI occurred with a concomitant reduction in the number of EPP. These two traits exhibited significant negative genetic correlations in all the three populations. EPP and GY were positively correlated. Senescence was negatively correlated with GY across the three populations under both the water regimes, whereas positive correlations between chlorophyll content (CEL and CYL) and GY were observed (Tables 3, 4), suggesting that SG traits were relevant under both the water regimes. The radiation use efficiency is higher in genotypes with high chlorophyll content, which reflects the ability of the plant to capture more solar energy for a longer period during grain filling when senescence is delayed. Drought stress during grain filling accelerates leaf senescence, and genotypes that maintain functional leaf areas are more capable of filling kernels. The increased chlorophyll content was reflected in an increase in EPP under drought stress. The genotypic correlations between EPP and CEL under WS conditions were significant and positive for all the three populations. Similarly, the correlations between chlorophyll content and GY were positive and stronger than those with leaf senescence, suggesting the merit of precision phenotyping over abstract visual scores, as also recommended by Masuka et al. (2012).

**Table 3:** Phenotypic correlations between grain yield and secondary traits under WW and WS conditions among the three biparental populations

Population	Treat.	Secondary traits							
		ASI	EPP	SENES	CEL	CYL	PH	EH	RC
CML444 × MALAWI	WW	−0.06ns	0.13ns	−0.11ns	0.40***	0.34***	0.37***	0.17ns	−0.27***
	WS	−0.51***	0.44***	−0.07ns	0.28***	0.20*	0.36***	0.26***	0.11ns
CML440 × CML504	WW	−0.18*	0.38***	−0.36***	0.37***	0.28**	0.40***	0.34***	0.17*
	WS	−0.28***	0.59***	−0.17**	0.30***	0.31***	0.33***	0.20**	0.01ns
CML444 × CML441	WW	−0.23**	0.44***	−0.32***	0.30***	0.29***	0.13*	0.12ns	−0.17*
	WS	−0.28***	0.46***	−0.46***	0.60***	0.50***	0.40***	0.44***	0.08ns

Abbreviations are given in Table 1

*, ** and *** significance levels at 5, 1 and 0.1 %, respectively, and *ns* non-significance

**Table 4:** Genotypic correlations between grain yield and secondary traits under WW and WS conditions among the three biparental populations

Population	Treat.	Traits							
		ASI	EPP	SENES	CEL	CYL	PH	EH	RC
CML444 × MALAWI	WW	−0.14ns	NA^ф^	−0.12ns	0.69***	0.53**	0.70***	0.28ns	
	WS	−0.89***	NA^ф^	−0.12ns	0.76***	0.59**	0.60***	0.40***	0.25ns
CML440 × CML504	WW	−0.42*	NA^ф^	−0.89***	0.86***	NA^ф^	0.61***	0.58***	NA^ф^
	WS	−0.86***	NA^ф^	−0.63***	NA^ф^	NA^ф^	0.86***	0.49**	0.05ns
CML444 × CML441	WW	−0.38*	0.87***	−0.71***	0.66***	0.79***	0.22ns	0.16ns	−0.28ns
	WS	−0.45***	0.77***	−0.83***	NA^ф^	0.79***	0.61***	0.63***	0.24ns

Abbreviations are given in Table 1

*, ** and *** significance level at 5, 1 and 0.1 %, respectively, and *ns* non-significance

Water stress slightly reduced the mean and average genetic variance of PH and EH (Table 2). In addition, the PH and EH were better correlated with GY under WS than under WW (Tables 3, 4). Under drought, tall plants apparently had a greater capacity for grain filling than shorter plants, most likely because of larger photosynthetically active leaf areas and greater stem reserves.

A significant correlation between GY and root capacitance was not observed under WS, and a weak association between these two traits was observed under WW (Tables 3, 4). Also, this trait recorded very low heritability estimates across the three populations, reflecting poor repeatability of the measurement (Table 2). Considering the poor correlation with GY and heritability estimates for root capacitance, the trait was not considered further for QTL mapping in this investigation.

### QTL mapping for secondary traits under contrasting water regimes

We detected a total of 203 QTL in individual QTL analyses for ASI, EPP, SENES, CEL, CYL, EH and PH, under both the water regimes, among the three populations with varying magnitudes (Tables S1, S2 and S3). However, approximately 65 % of the QTL were detected under WS, which confirms the importance and relevance of secondary traits in breeding for drought tolerance. Both the parental lines in each population contributed positive alleles for all the traits evaluated. QTL were detected almost on all the chromosomes for various secondary traits and interestingly only the region from chromosome 3 harboured QTL for all the morpho-physiological traits under WS and WW conditions (Tables S1, S2, S3, Figs. 1, S1). The QTL for GY and ASI under WW and WS in Mexico were previously reported by Almeida et al. (2013). In the section below, we have presented the QTL results for EPP, SENES, CEL, CYL, PH and EH. The QTL information from Almeida et al. (2013) was used to graphically visualise the co-location of secondary trait QTL with that of GY and ASI.

#### QTL for EPP

A total of 29 QTL were detected for EPP among all the three populations under both the water regimes. Narrow phenotypic variance for the trait resulted in smaller additive effects for most QTL detected. In the population CML440 × CML504, two genomic regions were detected under WW conditions on chromosome 1 (~64.26–76.05 and 90.77–90.78 Mb), and one region was detected under WS conditions on chromosome 5 (~206.33–208.90 Mb) (Table S2). In the CML444 × CML441 population, a QTL on chromosome 10 was detected (~99.47–120.54 Mb), which explained 32.3 % of the phenotypic variance under WS conditions (Table S3).

#### QTL for stay-green traits

The high correlation between SENES, CEL and CYL partially explained the co-localisation of QTL for these traits under WS. A total of 80 QTL were detected for SENES, CEL and CYL under both the water regimes among the three biparental populations (Tables S1, S2 and S3). Almost equal number of QTL was identified under WS and WW conditions for SENES. No QTL were detected under WW condition in RIL families of CML444 × MALAWI. Large-effect QTL were detected for SENES in all the three populations only under WS conditions, indicating the importance of this trait as a drought adaptation mechanism. A QTL on chromosome 4 (~104.16–128.63 Mb) was detected in the CML444 × MALAWI population that explained close to 16 % of the phenotype variance (Table S1). In the F_2:3_ populations, a QTL on chromosome 10 (~120.54–146.55 Mb) and two others on chromosome 2 (~195.55–195.93 Mb and 197.10–199.41 Mb) explained sizeable (~13 and ~21 %, respectively) phenotypic variance (Tables S2, S3). With regard to the chlorophyll content, a QTL on chromosome 3 (~52.80–210.16 Mb) was detected in the CML444 × CML441 population under WS conditions that explained around 16 and 21 % of the phenotypic variance for CEL and CYL, respectively (Table S3).

#### QTL for PH and EH

A total of 62 QTL were detected for PH and EH in all the three populations under WW and WS regimes. The QTL were located almost across all the chromosomes for both the traits. Interestingly, in all the three populations, QTL for EH were detected in the interval of 10.04–06 under both the water conditions (Tables S1, S2, S3, 5). Both parental lines in each population contributed with positive QTL alleles. CML444 contributed 55 and 70 % of favourable allele of PH and EH QTL in the two crosses, where it was the common parent (Tables S1, S3). With regard to CML440 × CML504, CML504 contributed 55 % of the favourable alleles for these two traits (Table S2). Most of the major QTL were detected under WW conditions. A QTL detected in the CML440 × CML504 population for PH under WW conditions on 1.08 (~217.50–239.31 Mb) explained around 31 % of the phenotype variance. Under WS conditions, a QTL on chromosome 5 (~97.98–167.87 Mb) was detected that explained around 11 % of the phenotypic variance in CML444 × CML441 for PH. Similarly, another region on chromosome 7 (~129.79–134.85 Mb) accounted for 11 % of phenotypic variance for PH under WS conditions in the CML440 × CML504 population.

**Table 5:** Meta-QTL for stay-green (SG), ears per plant (EPP) and plant-to-ear height ratio (PEH) traits across the three populations identified using across the population analysis

Trait	mQTL^a^	Bin	Pos. (cM)	Confidence interval (cM)	Flaking markers	Physical interval (Mb)	QTL number	QTL integrated^b^
SG	mQTL_SG_1a	1.03	129.14	120.05–138.23	pza02376.1-bnlg2238	44.36–55.08	4	*pop2CYL_WS1, pop3CEL_WW1b, pop3Sen_WW1b, pop3Sene_WS1b*
	mQTL_SG_1b	1.05/06	174.06	170.87–177.25	pza02741.1-phm5622.21	161.07–183.83	4	*pop2SenWW1a, pop3Sen_WW1a, pop3Sen_WS1b, pop3CYL_WS1a*
	mQTL_SG_3	3.06	101.3	98.49–104.0	pza02212.1-umc7	169.75–178.23	8	*pop3CYl_WS3, pop2SenWW3, pop3SenWW3, pop2CEl_WW3a, pop1CYl_WS3, popCEL_WS3, pop2CEL_WW3b, pop3CEl_WW3*
	mQTL_SG_4	4.09	98.6	92.52–104.66	pza00529.4-phm4310.112	240.77–244.08	4	*pop2CYL*-*WW4, pop1Sen_WS4b, pop3CYl_WS4, pop2CEl_WW4*
	mQTL_SG_5a	5.04	85.02	80.58–89.46	pzb01017.1-pza00148.3	158.03–164.23	6	*pop2CYl_WS5a, pop3Sen_WS5, pop3CEl_WS5, pop2Sen_WW5, pop2CEl_WW5, pop3CYl_WS5*
	mQTL_SG_5b	5.05	129.72	124.48–134.97	phm13696.11-pza01142.4	175.3–199.69	4	*pop2CYl_WS5b, pop3CYl*-*WW5, pop3CEl_WW5, pop2CYl_WW5*
	mQTL_SG_8	8.06	46.02	40.57–51.47	pmh15278.6-asg52a	155.48–159.76	4	*pop3Sen_WS8, pop1Sen_WS8, pop2Sen_WW8, pop2CEl_WW8*
	mQTL_SG_10	10.04/06	43.43	40.24–46.61	pza01919.2-pza03607.1	111.26–141.82	6	*pop3Sen_WS10, pop3CEl_WW10, pop3CYl_WW10a, pop3Sen_WW10, pop3CYl_WS10, pop1Sen_WS10,*
EPP	mQTL_EPP_2	2.08/09	116.46	115.20–117.72	pza02012.7-pza02727.1	218.28–227.92	3	*pop1EppWS1, pop2Epp_WW3, pop3Epp_WS2a*
	mQTL_EPP_3	3.06/07	100.2	96.05–104.3	pzd00027.2-umc63a	169.75–214.41	3	*pop1EppWW3, pop2Epp_WS3a, pop3EppWS3*
PEH	mQTL_PEH_1	1.05	164	161.22–166.77	csu1138.4-pza02741.1	119.01–161.08	6	*pop1PH_WS1, pop2PH_WW1a, pop2EH_WS1, pop2EH_WW, pop3EH_WS1, pop3EH_WW*
	mQTL_PEH_3	3.06	97.49	94.84–100.14	pza00186.4-phm17210.5	165.80–178.22	5	*pop1EH_WW_3, pop1WS3b, pop2PH_WS3, pop3PH_WS3, pop3EHWS3*
	mQTL_PEH_7	7.03	25.01	23.93–26.31	pzb00752.1-pza02854.13	131.10–137.83	7	*pop1EH_WW7, pop3EH_WS7, pop2PH_WS7, pop2EH_WS7, pop3PH_WS7, pop2EH_WW7, pop1PH_WW7*
	mQTL_PEH_10	10.05/06	69.8	58.14–81.46	npi232a-pza02527.2	130.59–148.48	4	*pop1EH_WS10, pop1EH_WW10, pop2EH_WS10, pop3EH_WW10b*

^a^Meta-QTL for stay-green (SG), ears per plant (EPP) and plant-to-ear height ratio (PEH) followed by the chromosome number

^b^Detected QTL using individual QTL analysis in each population in WW and WS conditions. The three populations were represented in the following order: pop1 (CML444 × MALAWI), pop2 (CML440 × CML504) and pop3 (CML444 × CML441). *Mb* megabase (10^6^ bp)

### Clusters of QTL detected by meta-analysis

Of the 203 QTL detected for all secondary traits under both the water regimes (Tables S1, S2 and S3), we plotted 174 onto a consensus map, developed from the three populations to perform a meta-QTL analysis (mQTL). The remaining QTL intervals that were not supported by a minimum of two anchor markers, and QTL explaining less than 2 % of the variance were excluded from the analysis. mQTL were declared only when it was common to all the three biparental populations or when one region harboured an elevated number of QTL derived from a minimum of two populations. In this study, we identified eight mQTL for SG traits, two for EPP and four for PEH, respectively, with a confidence interval of 95 % (Table 5), which are plotted in the consensus map (Fig. 1). Additionally, mQTL for GY and ASI were noted, as previously described (Almeida et al. 2013). mQTL for SG were distributed on chromosomes 1, 3, 4, 5, 8 and 10. Two mQTL each on chromosomes 1 and 5 were detected while the rest had one each. For EPP, mQTL were detected on chromosomes 2 and 3. mQTL for PEH were distributed on chromosomes 1, 3, 7 and 10. The confidence intervals for the 14 mQTL identified in this study ranged from 2.30 to 23.32 cM. These values were well below the previously established arbitrary threshold of 30 cM (Hund et al. 2011) for mQTL studies. In addition, we also provided the physical intervals of mQTL to be able to compare them with the previously reported results for use in marker-assisted breeding and the identification of candidate genes in these regions (Table 5). Considering only SG, the mQTL on chromosome 3 (mQTL_SG_3) had the largest number of QTL integrated from all the three populations under WW and WS conditions. For PEH, the mQTL on chromosome 7 (mQTL_PEH_7) harboured seven QTL under WW and WS conditions, and for EPP, both mQTL integrated the same number of QTL. All 14 mQTL for SG, EPP and PEH contained QTL derived from both the water regimes, indicating that those regions might play an important role in conferring a constitutive drought adaptation response to maize (Table 5).

Most of the mQTL for secondary traits overlapped with the previously identified adaptive or constitutive regions responsible for the GY detected across all the three populations (Almeida et al. 2013). Constitutive genomic regions regulating grain yield on chromosome 4 (~242.02–244.10 Mb) and 5 (~171.69–199.70 Mb) overlapped with mQTL_SG_4 and mQTL_SG_5b. The adaptive region on chromosome 7 (~123.61–132.28 Mb) overlapped with the mQTL for PEH, mQTL_PEH_7. Two genomic regions harboured mQTL for grain yield concomitant with SG and PEH traits. The first mQTL, located on bin 1.05/06 (~161.07–183.83 Mb), overlapped with the mQTL_SG_1b and mQTL_PEH_1. The second mQTL, located on chromosome 10 (~121.49–147.76 Mb), overlapped with mQTL_SG_10 and mQTL_PEH_10 (Table 5; Fig. 1). The mQTL on chromosome 3 (~169.75–178.23 Mb), reported by Almeida et al. (2013) as an important adaptive region regulating ASI under drought conditions, overlapped with the mQTL detected for all the secondary traits in this study (Table 5; Figs. 1, S1), indicating the importance of this genomic region for expression of adaptive and constitutive drought tolerance.

## Discussion

While the heritability estimates considerably decreased for GY under WS conditions, the secondary traits tended to have similar or substantially higher heritability estimates under WS than WW conditions, indicating their potential to aid in selection decisions when selections based on GY under WS alone may not be reliable due to the quality of the trial measurements. Enhanced genetic variance and heritability estimates of secondary morpho-physiological traits under drought conditions were also reported by Betrán et al. (2003) and Lu et al. (2011). Besides heritability, as suggested by Bänzinger et al. (2000), robust correlation of the secondary traits with GY is an important attribute that would enable their routine integration in the breeding programmes. In the current investigation, significant phenotypic and genotypic correlations were observed between GY on the one hand and ASI, EPP, SG and PEH on the other that are in agreement with the previous reports (Bolaños and Edmeads 1996; Ribaut et al. 1997; Messmer et al. 2009; Zheng et al. 2009; Lu et al. 2011). Of the secondary traits evaluated, ASI and EPP showed consistent positive associations with GY under WS conditions in all the three populations, which is noteworthy. SG that implies a reduction in the rate of leaf senescence during grain filling is one of the most visually distinctive traits between older and newer hybrids (Duvick et al. 2004). In our study, SG traits recorded moderate to high positive correlations with GY under both the water regimes, which is consistent with the observations of previous studies (Bänzinger et al. 2000; Zheng et al. 2009; Lu et al. 2011; Messmer et al. 2011).

Though a number of studies in the past have identified genomic regions responsible for GY and associated secondary traits under WS conditions (Beavis et al. 1994; Ribaut et al. 1997; Lima et al. 2006; Messmer et al. 2009; Zheng et al. 2009; Messmer et al. 2011), reports of their successful utilisation in the breeding programme are scarce. Population-specific nature of QTL, low-density marker maps and extensive LD decay in tropical maize germplasm are among the various possible reasons. Here, we identified a set of mQTL and a pair of flanking SNP markers for each mQTL for various secondary traits by detecting and combining QTL information from three key tropical biparental populations, which potentially enables marker-assisted selection for key secondary traits in the tropical breeding programmes, where drought stress is the most important constraint for enhanced productivity. Additionally, use of SNP markers with known physical positions enables objective comparison with the previously reported QTL that are associated with secondary traits. The co-location of the secondary traits QTL with that of GY partially explained the medium to high correlation observed between GY and secondary traits. Of the seven GY-mQTL detected by Almeida et al. (2013), five overlapped with at least one secondary trait in the current study (Table 5; Fig. 1).

While a number of mQTL for secondary traits integrated QTL across both the water regimes, ASI and EPP mQTL regions contained most of the QTL from WS conditions, which reinforces stress adaptive nature of these traits and also explains the enhanced correlation of GY with ASI and EPP, especially under WS conditions.

Two genomic regions on chromosome 1 and chromosome 10 harboured overlapping mQTL for GY, SG and PEH. The cluster on 1.05/1.06 contained mQTL for GY (161.07–183.29 Mb), SG (161.07–183.83 Mb) and PEH (119.01–161.08 Mb), while the cluster on chromosome 10 contained mQTL for GY (121.49–147.46 Mb), SG (111.26–141.82 Mb) and PEH (130.59–148.48 Mb). Many earlier studies have reported QTL for yield and secondary traits in these two regions on chromosome 1 and chromosome 10 under optimal and WS conditions. A meta-analysis by Li et al. (2010) involving 17 independent QTL mapping studies with grain yield, flowering traits and plant height revealed mQTL for drought tolerance on bin 1.05/06 at a physical interval of 178.87–180.72 Mb and on chromosome 10 in the interval of 116.24–126.70 Mb. Zheng et al. (2009) evaluated a F_2:3_ population (Qi-319 × Mo17) and reported a large-effect constitutive QTL for SG expressed at 40, 50 and 60 days after flowering at the same physical interval (164.55–200.72 Mb) in bin 1.06 under optimal conditions. Using 160 SSR markers in CML444 × MALAWI population, Messmer et al. (2011) detected a QTL for chlorophyll content in 1.06 with a marker peak at approximately 185.06 Mb under WW and WS conditions. Trachsel et al. (2010) evaluated the same population under greenhouse conditions and detected a QTL in 1.06 that was responsible for high leaf chlorophyll content and early vigour through enhanced efficiency of photosynthetic machinery (Φ_PSII_). More recently, Cai et al. (2012a) evaluated a population of RILs from a cross between Ye478 × Wu312 and detected a QTL on 10.04 that was responsible for the regulation of chlorophyll content in ear leaves under optimal, low nitrogen and low phosphorus levels. The mQTL on chromosome 10 was also confirmed by Messmer et al. (2011) for chlorophyll level and leaf senescence under drought and optimal conditions. Besides SG traits, the mQTL regions on chromosomes 1 and 10 have also been found to be constitutively regulating plant height under both the water regimes across temperate and tropical germplasm (Tang et al. 2007; Salvi et al. 2011; Chen et al. 2011; Cai et al. 2012a, b). Notably, most of the studies reported the QTL for SG traits on chromosome 10 (10.04–07) to be of large effect (*R* ^2^ > 10 %), which indicates potential for marker-assisted introgression in future in pedigree and back-cross-based line improvement programmes.

The two mQTL regions identified on 4.09 and 5.05 for SG and GY predominantly integrated QTL under WS conditions, indicating stress adaptive nature of SG traits. These regions have been previously reported to be significantly associated with leaf senescence (Messmer et al. 2011), maintenance of leaf green area during post-flowering (Wang et al. 2012) and stay-green (Zheng et al. 2009).

One of the most important genomic regions uncovered in this study was on chromosome 3 between 169.75 and 178.28 Mb (3.06), which harboured a number of QTL for most of the secondary traits considered here (Fig. S1). Considering the fact that the secondary traits were not correlated well among themselves, identification of several QTL in this interval for ASI, EPP, SG and PEH suggests possibility of cluster of tightly linked loci orchestrating drought tolerance through coordinated expression of several secondary traits. Interestingly, 65 % of the integrated QTL in this region were detected under WS conditions, which emphasised the predominantly adaptive nature of this region for drought tolerance. A number of studies in the past have reported QTL in this region for one or several traits associated with drought tolerance. Zheng et al. (2009) reported a QTL in 3.06 between 175.78 and 194.18 Mb that was responsible for the regulation of SG traits at 40 days after flowering time. Similarly, Messmer et al. (2011) reported a QTL on 3.06 for leaf senescence under intermediary water stress and Cai et al. (2012a, b) detected a QTL between 169.61 and 190.25 Mb for chlorophyll content in maize under low nitrogen levels and optimal conditions. Evaluating a population of BC5F4 individuals obtained by crossing Gáspe Flint (open-pollinated variety as donor) with B73, Salvi et al. (2011) identified a QTL in 3.05–0.7 that was important for the regulation of EPP and plant height. Besides the above, three meta-QTL studies found genomic regions overlapping with 3.06 to be associated with drought tolerance. Hao et al. (2010) and Li et al. (2010) identified mQTL on 3.06, between 172.2 and 176.2 Mb by combining information from twelve and seven independent studies, respectively, that was associated with drought tolerance. Another meta-analysis integrating 15 individual studies under optimal and drought conditions (Hund et al. 2011) revealed the significance of this region (3.06) in regulating expression of root-related traits such as root pulling force and lateral root extension that are positively associated with drought tolerance. Besides these meta-analyses, a genome-wide association study involving leading inbred lines of popular Chinese maize hybrids identified a SNP (pzb01919.1) in 3.06 at 178.23 Mb that was strongly associated with GY, ASI and the drought tolerance index across different environments (Hao et al. 2011).

The physical interval delimited on 3.06, which contained mQTL for ASI, SG, EPP and PEH harboured two interesting candidate genes viz., *Zmm16* (GRMZM2G110153—MADS-domain transcription factor), which was implicated in reproductive organ development (Setter et al. 2011), and *psbs1* (GRMZM2G077333_T01—photosystem II subunit), which acts as a pigment chaperone for the incorporation of chlorophyll molecules into pigment-binding proteins during photoassimilate production (Hankamer and Barber 1997).

Six of the mQTL regions identified by Almeida et al. (2013) across the three populations overlapped with at least one secondary trait in this study, indicating their positive association and potential utilisation in marker assisted pyramiding of complementary QTL towards enhanced drought tolerance in tropical maize germplasm. Saturating the physical intervals of ten mQTL regions identified in the current study with higher density of markers and identifying donor-specific haplotypes may facilitate large-scale application of MAS of these regions across diverse genetic backgrounds in future.

## Electronic supplementary material

Below is the link to the electronic supplementary material.

Supplementary material 1 (DOCX 266 kb)
