# Supplementary material for: Molecular mapping across three populations reveals a QTL hotspot region on chromosome 3 for secondary traits associated with drought tolerance in tropical maize
Source: Mol Breed. 2014 Mar 16;34(2):701–15. doi: 10.1007/s11032-014-0068-5 (PMC4092235; doi:10.1007/s11032-014-0068-5)
Supplement: Supplementary file 1 — Supplementary material 1 (DOCX 266 kb) [file 11032_2014_68_MOESM1_ESM.docx]

**Supplementary information:**

**Table S1.** Genomic positions, LOD values, genetic effects, gene actions and phenotypic variance (R^2^) for detected QTL in WW and WS environments on RILs of CML444xMALAWI. Abbreviations are given in Table 1.

| **Trait** | **Treat.** | **Chr** | **Pos (cM)** | **Marker Interval** | **Physical position**^1^ | **LOD** | **R^2^** | **Add**^2^ | **Direction** |
| --- | --- | --- | --- | --- | --- | --- | --- | --- | --- |
| ASI | WW | **-** | **-** | **-** | **-** | **-** | **-** | **-** | **-** |
|  | WS | 1 | 267.0 | d8.3-bnlg1720 | 265.20-264.73 | 3.56 | 6.01 | -0.37 | MALAWI |
|  |  | 3 | 173.0 | umc7-umc3b | 170.27-192.26 | 4.55 | 7.98 | -0.42 | MALAWI |
|  |  | 5 | 172.0 | pza02209.2-mc48b | 180.43-** | 2.86 | 5.50 | -0.36 | MALAWI |
|  |  | 10 | 126.0 | bnlg236-umc1038 | 140.96- 148.09 | 3.35 | 10.67 | 0.44 | CML444 |
| EPP | WW | 1 | 336.0 | pza02359.10-phm9807.9 | 293.91-294.31 | 3.78 | 8.43 | 0.02 | CML444 |
|  |  | 3 | 95.0 | phi053-bnlg420 | 126.51-143.17 | 3.65 | 7.25 | 0.02 | CML444 |
|  | WS | 1 | 346.0 | bnl6.32-bnlg2123 | 297.96-292.60 | 3.29 | 8.33 | -0.03 | MALAWI |
|  |  | 2 | 179.0 | umc8g-umc135 | 40.94-41.69 | 3.38 | 7.67 | 0.03 | CML444 |
| SENES | WW | - | - | - | - | - | - | - | - |
|  | WS | 4 | 76.0 | pza03231.1-pza03409.1 | 104.16-128.63 | 7.89 | 15.76 | -0.30 | MALAWI |
|  |  | 4 | 85.0 | csu100-umc156a | 136.06-150.21 | 4.66 | 8.52 | 0.22 | CML444 |
|  |  | 8 | 114.0 | phm12749.13-umc48a | 155.07-161.78 | 4.25 | 8.01 | 0.21 | CML444 |
|  |  | 10 | 67.0 | pza00048.1-pza01919.2 | 98.58-111.26 | 3.44 | 6.92 | -0.21 | MALAWI |
| CEL | WW | 9 | 127.0 | umc113a-umc105a | 17.81-18.56 | 4.03 | 7.85 | 0.27 | CML444 |
|  | WS | 6 | 233.0 | phm4468.13- umc2059 | 167.53-187.77 | 4.24 | 9.46 | -0.21 | MALAWI |
| CYL | WW | 9 | 128.0 | umc113a-umc105a | 17.81-18.56 | 2.63 | 5.89 | 0.23 | CML444 |
|  | WS | 3 | 155.0 | umc7- pza03733.1 | 165.99-180.53 | 2.70 | 5.84 | -0.17 | MALAWI |
| EH | WW | 3 | 155.0 | pza03733.1-umc7 | 170.27-180.53 | 5.67 | 9.47 | 2.46 | CML444 |
|  |  | 4 | 98.0 | umc156a-pza00453.2 | 150.21-166.28 | 3.50 | 6.49 | 2.09 | CML444 |
|  |  | 6 | 233.0 | umc2059-phm4468.13 | 167.53-168.77 | 4.09 | 6.63 | 2.05 | CML444 |
|  |  | 7 | 10.0 | phm4135.15-umc1066 | 6.44-10.79 | 3.19 | 5.52 | 1.87 | CML444 |
|  |  | 9 | 23.0 | umc1733-bnl1588 | 145.34-146.52 | 5.73 | 8.17 | 2.29 | CML444 |
|  |  | 9 | 105.0 | pza03416.7-umc105a | 16.98-18.56 | 2.82 | 4.48 | 1.69 | CML444 |
|  |  | 10 | 79.0 | umc1115-npi232a | 126.260-130.96 | 3.26 | 4.76 | -1.80 | MALAWI |
|  | WS | 6 | 152.0 | mmc0241- bnlg1732 | 145.49-151.96 | 3.80 | 5.68 | -2.14 | MALAWI |
|  |  | 6 | 214.0 | bnlg1740- umc2059 | 164.86-168.77 | 3.21 | 5.06 | 1.88 | CML444 |
|  |  | 10 | 66.0 | pza00048.1-pza01919.2 | 98.58-111.26 | 5.14 | 7.88 | -2.46 | MALAWI |
| PH | WW | 1 | 73.0 | bnlg1627-umc11a | 18.92-34.92 | 2.98 | 5.26 | -2.59 | MALAWI |
|  |  | 1 | 156.0 | bnlg1057-umc1122 | 191.01-201.47 | 2.79 | 5.47 | 2.64 | CML444 |
|  |  | 6 | 70.0 | pza03069.4-bnl2151 | 81.81-88.70 | 5.15 | 10.18 | -3.62 | MALAWI |
|  |  | 7 | 116.0 | pza00795.1-pza02373.1 | 159.42-161.62 | 6.11 | 12.03 | 3.96 | CML444 |
|  |  | 8 | 39.0 | npi110a-umc103a | 10.19-16.95 | 2.55 | 3.80 | -2.20 | MALAWI |
|  | WS | 1 | 88.0 | pza00962.1- bnlg439 | 43.83-43.86 | 4.78 | 8.86 | -2.65 | MALAWI |
|  |  | 6 | 48.0 | umc85a- bnlg426 | 8.29-16.25 | 4.20 | 7.89 | -2.53 | MALAWI |
|  |  | 8 | 53.0 | bnlg669-phm2350.17 | 22.66-23.99 | 2.57 | 5.39 | -2.13 | MALAWI |

^1^Physical position of the marker flanking the QTL in Mb (10^6^ pb). ^2^QTL with additive effects are presented with positive values and were contributed by the parent CML444; the negative values were derived from the parent MALAWI. ** Unknown physical position.

**Table S2**. Genomic positions, LOD values, genetic effects, gene actions and phenotypic variations (R^2^) for detected QTL in well-watered (WW) and water-stressed (WS) environments on F_2:3_ families from CML440xCML504. Abbreviations are given in Table 1.

| **Trait** | **QTL Position** | | | | | **LOD** | **Genetic Effect**^2^ | | | **Gene Action**^3^ | |  |
| --- | --- | --- | --- | --- | --- | --- | --- | --- | --- | --- | --- | --- |
|  | **Treat.** | **Chr.** | **Pos. (cM)** | **Marker Interval** | **Physical position** ^1^ |  | **R^2^ (%)** | **Add** | **Dom** | **\|d/a\|** | **Nature** | **Direction** |
| ASI | WW | 1 | 653.0 | phm1438.34-pza03578.1 | 212.39 - 252.22 | 3.14 | 12.87 | 0.30 | -0.40 | 1.34 | D | CML440 |
|  |  | 2 | 124.0 | pza01336.1-phm4880.179 | 31.39 - 103.49 | 2.70 | 9.12 | -0.34 | -0.12 | 0.46 | PD | CML504 |
|  |  | 9 | 90.0 | pza00947.1-pzb01899.1 | 96.89 - 98.51 | 3.72 | 6.44 | 0.32 | -0.16 | 0.49 | PD | CML440 |
|  | WS | 1 | 136.0 | pza00887.1-pza03521.1 | 10.07-10.93 | 2.59 | 3.50 | 0.26 | -0.18 | 0.70 | PD | CML440 |
|  |  | 2 | 69.0 | phm6111.5-pza00590.1 | 21.98-29.99 | 5.99 | 8.10 | 0.30 | -0.43 | 1.40 | OD | CML440 |
|  |  | 3 | 148.0 | phm2290.12-phm15449.10 | 121.88-125.08 | 3.34 | 3.42 | -0.06 | 0.37 | 6.24 | OD | CML504 |
|  |  | 5 | 270.0 | phm3512.186-pza02099.3 | 203.43-206.33 | 5.20 | 6.57 | -0.40 | -0.17 | 0.42 | PD | CML504 |
|  |  | 7 | 161.0 | pza01542.1-pza02449.13 | 129.79-134.85 | 3.12 | 1.99 | 0.09 | 0.27 | 2.90 | OD | CML440 |
|  |  | 8 | 96.0 | pza00717.15-pza01257.1 | 68.48-68.79 | 3.34 | 4.41 | 0.35 | -0.01 | 0.03 | A | CML440 |
|  |  | 9 | 118.0 | pza02397.1-phm4905.6 | 133.88-133.92 | 3.80 | 5.18 | -0.39 | 0.04 | 0.11 | A | CML504 |
|  |  | 9 | 168.0 | pza00323.3-pza00832.1 | 142.50-147.13 | 3.21 | 3.82 | 0.25 | -0.21 | 0.83 | PD | CML504 |
| EPP | WW | 1 | 252.0 | pza03189.4-pza01267.3 | 64.26-76.05 | 7.90 | 14.18 | -0.01 | -0.04 | 4.11 | OD | CML504 |
|  |  | 1 | 275.0 | pza03240.1-pza03240.2 | 90.77-90.78 | 8.14 | 14.14 | 0.01 | 0.04 | 4.19 | OD | CML440 |
|  |  | 2 | 69.0 | phm6111.5-pza00590.1 | 21.98-21.99 | 3.45 | 2.29 | 0.01 | -0.01 | 0.60 | PD | CML440 |
|  |  | 6 | 171.0 | pzb01222.1-pza02815.25 | 164.42-167.88 | 4.15 | 5.82 | 0.01 | -0.01 | 1.21 | D | CML440 |
|  |  | 7 | 164.0 | pza01542.1-pza02449.13 | 129.79-134.85 | 2.89 | 1.73 | 0.01 | 0.01 | 1.61 | OD | CML440 |
|  | WS | 1 | 173.0 | pza02094.9-pza01030.1 | 15.72-17.68 | 4.87 | 5.59 | 0.05 | -0.23 | 4.42 | OD | CML440 |
|  |  | 1 | 229.0 | pza02376.1-phm2130.29 | 44.51-55.56 | 4.28 | 4.29 | 0.05 | -0.21 | 4.62 | OD | CML440 |
|  |  | 1 | 567.0 | PZB00008.1-pza01588.1 | 258.50-268.37 | 7.66 | 8.73 | -0.03 | 0.31 | 10.37 | OD | CML504 |
|  |  | 3 | 230.0 | PZB01457.1-phm13742.5 | 212.72-213.61 | 4.77 | 5.41 | -0.05 | -0.24 | 4.96 | OD | CML504 |
|  |  | 3 | 267.0 | pza01154.1-pza01688.3 | 216.03-223.67 | 2.90 | 3.64 | 0.01 | 0.20 | 15.54 | OD | CML440 |
|  |  | 5 | 236.0 | pza00963.3-phm3512.186 | 203.43-207.27 | 4.04 | 4.69 | -0.02 | -0.24 | 15.73 | OD | CML504 |
|  |  | 5 | 294.0 | pza02099.3-pza01680.3 | 206.33-208.90 | 11.04 | 14.22 | 0.18 | 0.34 | 1.90 | OD | CML440 |
|  |  | 7 | 98.0 | phm10225.15-phm1912.20 | 155.97-162.17 | 3.58 | 3.96 | 0.06 | 0.21 | 3.61 | OD | CML440 |
|  |  | 7 | 134.0 | pza00405.6-pza03166.1 | 137.63-138.55 | 4.45 | 4.77 | -0.06 | -0.23 | 3.58 | OD | CML504 |
|  |  | 10 | 156.0 | pza01141.1-phm3844.14 | 120.54-146.55 | 3.26 | 4.47 | -0.15 | 0.08 | 0.53 | OD | CML504 |
| SENES | WW | 1 | 151.0 | pza03521.1-pza0355.1 | 10.07-12.21 | 4.14 | 6.46 | -2.31 | -0.47 | 0.20 | A | CML504 |
|  |  | 1 | 530.0 | pza03020.8-phm3563.17 | 284.03-282.04 | 3.00 | 4.66 | 1.06 | 1.83 | 1.73 | O | CML440 |
|  |  | 3 | 184.0 | pza02299.16-pza00920.1 | 103.38-142.82 | 3.23 | 5.12 | 1.80 | 0.34 | 0.19 | A | CML440 |
|  |  | 5 | 115.0 | phm565.31-phm13675.17 | 24.24-66.81 | 3.62 | 4.46 | 1.58 | -1.14 | 0.72 | PD | CML440 |
|  |  | 6 | 170.0 | pzb01222.1-pza02815.25 | 164.4-167.88 | 2.74 | 2.29 | -0.97 | 0.87 | 0.89 | D | CML504 |
|  |  | 7 | 9.0 | pza00418.2-pza01210.1 | 71.72-75.10 | 2.82 | 4.29 | 0.66 | -1.84 | 2.77 | OD | CML440 |
|  |  | 8 | 129.0 | pza00739.1-pza01049.1 | 105.80-129.04 | 3.37 | 5.50 | 2.16 | -0.04 | 0.02 | A | CML440 |
|  |  | 9 | 173.0 | pza00323.3-pza00832.1 | 142.50-147.13 | 3.58 | 5.21 | 0.69 | 2.39 | 3.44 | OD | CML440 |
|  | WS | 1 | 606.0 | pza00381.4-phm14475.7 | 237.64-245.118 | 2.75 | 4.37 | -1.88 | 0.82 | 0.44 | PD | CML504 |
|  |  | 3 | 43.0 | pza03527.1-pza02098.2 | 5.70-8.11 | 3.96 | 6.11 | 2.26 | -0.22 | 0.10 | A | CML440 |
|  |  | 9 | 48.0 | zhd1.1-pza01999.3 | 22.04-23.22 | 3.29 | 4.38 | -1.68 | 0.95 | 0.57 | PD | CML504 |
|  |  | 10 | 145.0 | pza01141.1-phm3844.14 | 120.54-146.55 | 3.36 | 17.81 | -3.82 | -5.78 | 1.51 | OD | CML504 |
| CEL | WW | 3 | 192.0 | pza00186.4-pza01962.12 | 165.80-178.23 | 6.02 | 9.74 | -49.29 | 3.05 | 0.06 | A | CML504 |
|  |  | 3 | 220.0 | pza03458.1-pzb01457.1 | 203.32-212.73 | 5.50 | 9.16 | 49.84 | -8.21 | 0.16 | A | CML440 |
|  |  | 4 | 44.0 | pza00436.7-phm2159.8 | 6.40-28.98 | 5.39 | 8.99 | -1.84 | 66.57 | 36.08 | OD | CML504 |
|  |  | 5 | 114.0 | phm565.31-phm13675.17 | 24.24-66.81 | 4.25 | 5.69 | -4.06 | 52.57 | 12.95 | OD | CML504 |
|  |  | 8 | 168.0 | phm4203.11-phm4757.14 | 133.53-151.45 | 3.37 | 4.43 | 20.64 | -29.29 | 1.42 | OD | CML440 |
|  | WS | 4 | 52.0 | pza03385.1-phm14717.2 | 37.0-40.52 | 4.27 | 7.03 | 19.82 | -9.97 | 0.50 | PD | CML440 |
|  |  | 6 | 133.0 | phm4503.25-phm2108.61 | 161.13-161.66 | 3.28 | 5.08 | -16.44 | 8.99 | 0.55 | PD | CML504 |
| CYL | WW | 4 | 114.0 | pza03205.1-pza01810.2 | 202.88-203.77 | 3.92 | 2.57 | 18.00 | -0.99 | 0.05 | A | CML440 |
|  |  | 5 | 202.0 | phm13696.9-phm13696.11 | 175.36-175.37 | 2.53 | 3.72 | -4.37 | -29.52 | 6.76 | OD | CML504 |
|  |  | 7 | 162.0 | pza01542.1-pza02449.13 | 129.79-134.85 | 2.94 | 2.03 | 2.91 | 23.83 | 8.18 | OD | CML440 |
|  | WS | 1 | 252.0 | pza03189.4-pza01267.3 | 64.26-76.05 | 2.80 | 4.69 | -15.49 | -5.04 | 0.33 | PD | CML504 |
|  |  | 5 | 176.0 | pza01530.1-pza02408.2 | 37.79-180.41 | 3.82 | 5.00 | 14.92 | -3.05 | 0.20 | A | CML440 |
|  |  | 5 | 230.0 | pza00980.1-pza00963.3 | 203.77-207.27 | 3.12 | 5.38 | -17.67 | 0.58 | 0.03 | A | CML504 |
|  |  | 7 | 164.0 | pza01542.1-pza02449.13 | 129.79-134.85 | 3.48 | 4.24 | -1.48 | 21.53 | 14.57 | OD | CML504 |
| EH | WW | 1 | 190.0 | pza01030.1-phm13619.5 | 17.68-22.03 | 2.88 | 4.00 | -2.76 | 0.11 | 0.04 | A | CML504 |
|  |  | 2 | 126.0 | pza01336.1-phm4880.179 | 31.39-103.49 | 6.72 | 10.47 | 0.35 | -6.08 | 17.48 | OD | CML440 |
|  |  | 7 | 169.0 | pza01542.1-pza02449.13 | 129.79-134.85 | 3.80 | 10.56 | 0.68 | 7.44 | 10.99 | OD | CML440 |
|  | WS | 1 | 189.0 | pza01030.1-phm13619.5 | 17.68-22.28 | 7.08 | 9.61 | -2.92 | 0.45 | 0.15 | A | CML504 |
|  |  | 5 | 153.0 | pza00996.1-pza01530.1 | 37.78-37.79 | 3.13 | 3.72 | -0.48 | -2.34 | 4.85 | OD | CML504 |
|  |  | 6 | 93.0 | pzb00414.2-phm15665.22 | 131.40-137.48 | 3.38 | 3.66 | 1.99 | 0.68 | 0.34 | PD | CML440 |
|  |  | 7 | 164.0 | pza01542.1-pza02449.13 | 129.79-134.85 | 3.69 | 6.21 | 0.43 | 3.49 | 8.14 | OD | CML440 |
|  |  | 9 | 79.0 | pza01791.2-pza00947.1 | 77.46-96.89 | 3.25 | 3.50 | -1.83 | -0.36 | 0.20 | A | CML504 |
|  |  | 10 | 146.0 | pza01141.1-phm3844.14 | 120.54-146.55 | 2.50 | 4.13 | -2.20 | 0.96 | 0.44 | PD | CML504 |
| PH | WW | 1 | 201.0 | phm13619.5-phm4597.14 | 22.28-38.61 | 4.87 | 8.52 | -4.91 | 0.84 | 0.17 | A | CML504 |
|  |  | 1 | 612.0 | pza02655.9-phm297.18 | 217.50-239.31 | 17.01 | 30.45 | -2.37 | 11.51 | 4.86 | OD | CML504 |
|  |  | 7 | 49.0 | pza02018.1-pza03583.1 | 86.40-128.40 | 3.04 | 4.85 | 3.47 | 3.52 | 1.01 | D | CML440 |
|  |  | 8 | 97.0 | phm4552.6-pza01257.1 | 67.93-68.79 | 4.79 | 7.38 | 4.95 | 0.46 | 0.09 | A | CML440 |
|  | WS | 1 | 199.0 | phm13619.5-phm4597.14 | 22.28-38.61 | 3.44 | 4.52 | -2.37 | -0.48 | 0.20 | A | CML504 |
|  |  | 1 | 571.0 | pza01588.1-pzb00008.1 | 258.50-268.37 | 5.02 | 6.84 | -2.74 | 1.37 | 0.50 | PD | CML504 |
|  |  | 3 | 187.0 | pza00920.1-pza00186.4 | 142.82-165.80 | 3.83 | 5.81 | -2.41 | 1.53 | 0.63 | PD | CML504 |
|  |  | 6 | 94.0 | pzb00414.2-phm15665.22 | 131.40-137.48 | 3.77 | 4.51 | 2.51 | -0.47 | 0.19 | A | CML440 |
|  |  | 8 | 102.0 | phm4552.6-pza02683.1 | 67.93-90.84 | 4.29 | 5.92 | 2.90 | -0.55 | 0.19 | A | CML440 |

^1^Physical position of the marker flanking the QTL expressed in Mb (10^6^pb). ^2^Genetic effects of the QTL are determined by the A: additives and D: dominant effects. QTL with additive effects are shown with positive values and were contributed by the parent CML440, and QTL with negative values are from the parent CML504. ^3^Gene action determined on the basis of the level of dominance was calculated using the ratio between dominant and additives effects of the QTL (|d/a|) according to Stuber et al. (1987) criterion: additive (A) = 0 – 0.20; partial dominance (PD) = 0.21 – 0.80; dominance (D) = 0.81 – 1.20, and overdominance OD > 1.20.

**Table S3**. Genomic positions, LOD values, genetic effects, gene actions and phenotypic variations (R^2^) for detected QTL in well-watered (WW) and water-stressed (WS) environments on F_2:3_ families from CML444xCML441. Abbreviations are given in Table 1.

| **Trait** | **QTL Position** | | | | | **LOD** | **Genetic Effect**^2^ | | | **Gene Action**^3^ | |  |
| --- | --- | --- | --- | --- | --- | --- | --- | --- | --- | --- | --- | --- |
|  | **Treat.** | **Chr.** | **Pos. (cM)** | **Marker Interval** | **Physical position** ^1^ |  | **R^2^ (%)** | **Add** | **Dom** | **\|d/a\|** | **Nature** | **Direction** |
| ASI | WW | 1 | 145.0 | pza03578.1-d8.2 | 252.22-265.19 | 2.57 | 1.85 | -0.10 | 0.18 | 1.80 | OD | CML441 |
|  |  | 1 | 571.0 | phm595.30-pza02087.2 | 281.82-284.06 | 3.28 | 4.94 | -0.15 | -0.16 | 1.05 | D | CML441 |
|  |  | 3 | 114.0 | phm2423.33-pza00297.2 | **-227.68 | 4.62 | 7.95 | -0.21 | -0.29 | 1.38 | OD | CML441 |
|  |  | 4 | 280.0 | pza02027.1-pza03459.1 | 132.98-134.29 | 2.66 | 6.27 | -0.15 | -0.32 | 2.14 | OD | CML441 |
|  |  | 5 | 165.0 | pza00963.3-pza02015.11 | 207.27-207.46 | 2.87 | 4.33 | -0.19 | 0.00 | 0.01 | A | CML441 |
|  |  | 7 | 63.0 | pza01909.2-pza01210.1 | 6.44-75.09 | 3.38 | 3.30 | -0.15 | 0.17 | 1.13 | D | CML441 |
|  |  | 10 | 96 | pza01001.2-phm3736.11 | 146.54-147.76 | 3.38 | 9.20 | 0.26 | -0.19 | 0.73 | PD | CML444 |
|  | WS | 1 | 117.0 | d8.2-pzb00114.1 | 265.20-275.98 | 2.54 | 1.81 | -0.04 | 0.29 | 7.06 | OD | CML441 |
|  |  | 1 | 571.0 | phm595.30-pza02087.2 | 281.81-284.05 | 4.24 | 4.74 | -0.35 | 0.08 | 0.22 | A | CML441 |
|  |  | 2 | 62.0 | pza01280.2-phm3668.12 | 149.43-195.55 | 4.45 | 6.76 | 0.44 | -0.08 | 0.19 | A | CML444 |
|  |  | 2 | 262.0 | pza01232.1-pza02939.10 | 155.86-157.15 | 14.07 | 20.20 | -0.68 | 0.12 | 0.18 | A | CML441 |
|  |  | 3 | 275.0 | pza01154.1-phm2672.19 | 216.03-219.86 | 2.57 | 2.07 | -0.16 | 0.31 | 1.93 | OD | CML441 |
|  |  | 4 | 182.0 | phm687.25-phm2159.8 | 17.48-28.98 | 3.76 | 5.10 | 0.40 | -0.13 | 0.33 | PD | CML444 |
|  |  | 6 | 48.0 | phm15961.13-pza00355.2 | 9.56-78.75 | 2.80 | 3.52 | -0.29 | -0.19 | 0.66 | PD | CML441 |
|  |  | 6 | 94.0 | pza00214.1-phm12794.47 | 91.70-128.47 | 2.99 | 3.43 | 0.28 | -0.28 | 1.00 | D | CML444 |
|  |  | 7 | 60.0 | pza01909.2-pza01210.1 | 6.43-75.09 | 2.76 | 1.63 | -0.10 | 0.32 | 3.21 | OD | CML441 |
|  |  | 10 | 40.0 | pza03605.1-phm5435.25 | 141.83-144.234 | 2.99 | 4.12 | -0.02 | -0.47 | 30.45 | OD | CML441 |
|  |  | 10 | 272.0 | phm5740.9-pzb01301.5 | 8.77-9.75 | 2.96 | 2.57 | 0.27 | 0.09 | 0.35 | PD | CML444 |
| EPP | WW | 1 | 483.0 | pza03183.5-pza03189.4 | 46.07-64.26 | 2.71 | 3.02 | 0.00 | -0.02 | 5.20 | OD | CML444 |
|  |  | 3 | 272.0 | pza01154.1-phm2672.19 | 219.03-219.86 | 2.74 | 3.31 | 0.00 | 0.02 | 4.75 | OD | CML441 |
|  |  | 9 | 42.0 | pzb01110.6-pza01062.1 | 24.03-88.06 | 4.41 | 9.50 | -0.01 | 0.03 | 3.33 | OD | CML441 |
|  |  | 10 | 273.0 | phm5740.9-pzb01301.5 | 8.77-9.75 | 2.73 | 2.88 | -0.01 | 0.01 | 1.03 | D | CML441 |
|  | WS | 2 | 184.0 | pza02264.5-phm13440.13 | 2.52-3.16 | 2.53 | 2.64 | 0.01 | 0.00 | 0.34 | PD | CML444 |
|  |  | 2 | 348.0 | pza01352.5-pza02170.1 | 226.45-231.19 | 3.22 | 3.83 | -0.01 | -0.02 | 3.33 | OD | CML441 |
|  |  | 3 | 116.0 | phm2423.33-pza00297.2 | **-227.68 | 2.99 | 1.59 | 0.00 | 0.01 | 6.50 | OD | CML444 |
|  |  | 4 | 125.0 | pza01905.12-phm2438.28 | 3.54-244.09 | 2.71 | 1.18 | -0.01 | 0.01 | 1.57 | OD | CML441 |
|  |  | 8 | 15.0 | pza01857.1-pza01079.1 | 14.12-156.10 | 4.67 | 5.19 | 0.01 | 0.01 | 0.63 | PD | CML444 |
|  |  | 10 | 236.0 | pza02398.2- pza01141.1 | 99.47-120.54 | 4.66 | 32.28 | -0.05 | 0.04 | 0.78 | PD | CML441 |
| SENES | WW | 1 | 115.0 | d8.2-pzb00114.1 | 265-19-275.98 | 2.63 | 1.74 | 1.59 | 1.08 | 0.68 | PD | CML444 |
|  |  | 1 | 345.0 | pza03200.2-pza02741.1 | 148.69-161.07 | 5.13 | 7.74 | 3.10 | 0.41 | 0.13 | A | CML444 |
|  |  | 1 | 425.0 | pzb02058.1-pza02195.1 | 28.52-39.29 | 3.31 | 3.92 | -2.25 | 0.07 | 0.03 | A | CML441 |
|  |  | 3 | 212.0 | pza00210.9-phm5502.31 | 29.69-67.28 | 3.15 | 4.48 | -2.35 | 0.08 | 0.03 | A | CML441 |
|  |  | 4 | 307.0 | fea2.3-pza02194.1 | 132.73-180.31 | 2.55 | 2.13 | 1.69 | 0.79 | 0.47 | PD | CML444 |
|  |  | 6 | 52.0 | phm15961.13-pza00355.2 | 9.56-78.75 | 4.88 | 7.40 | -3.00 | -0.44 | 0.15 | A | CML441 |
|  |  | 7 | 57.0 | pza01909.2-pza01210.1 | 6.43-75.09 | 3.95 | 7.61 | -1.08 | 4.59 | 4.24 | OD | CML441 |
|  |  | 10 | 101.0 | pza01001.2-phm3736.11 | 146.54-147.76 | 2.61 | 2.80 | 1.26 | 2.10 | 1.67 | OD | CML444 |
|  | WS | 1 | 15.0 | phm14475.7-phm174.13 | 256.24-294.91 | 3.86 | 3.70 | 0.78 | -3.33 | 4.26 | OD | CML444 |
|  |  | 1 | 192.0 | phm3034.3-pza01921.19 | 61.31-255.55 | 2.65 | 2.18 | 1.69 | 1.76 | 1.04 | D | CML444 |
|  |  | 1 | 344.0 | pza03200.2-pza02741.1 | 148.69-161.07 | 3.82 | 3.77 | 2.29 | -0.30 | 0.13 | A | CML444 |
|  |  | 2 | 90.0 | phm3668.12-phm7953.11 | 195.55-195.93 | 10.97 | 13.25 | -3.62 | 3.82 | 1.06 | D | CML441 |
|  |  | 2 | 295.0 | pza02731.1-phm16125.47 | 197.10-199.41 | 18.20 | 21.04 | 4.93 | -0.31 | 0.06 | A | CML444 |
|  |  | 5 | 137.0 | pza02015.11-pza03339.2 | 207.46-210.89 | 4.09 | 4.42 | 0.59 | 3.34 | 5.62 | OD | CML444 |
|  |  | 8 | 292.0 | phm1834.47-pza01316.1 | 162.44-164.37 | 2.62 | 2.64 | 1.96 | 0.29 | 0.15 | A | CML444 |
|  |  | 10 | 52.0 | pza03605.1-pza03603.1 | 141.82-141.83 | 2.93 | 3.76 | 0.78 | 2.78 | 3.58 | OD | CML444 |
| CEL | WW | 1 | 46.0 | pzb01227.6-pza00623.3 | 288.44-293.63 | 2.55 | 1.96 | 15.71 | -28.07 | 1.79 | OD | CML444 |
|  |  | 1 | 374.0 | phm12323.17-csu1138.4 | 53.35-119.02 | 2.88 | 3.35 | -20.24 | -39.14 | 1.93 | OD | CML441 |
|  |  | 3 | 115.0 | pza00297.2-phm2423.33 | **-227.68 | 2.91 | 1.58 | -2.72 | 30.46 | 11.18 | OD | CML441 |
|  |  | 4 | 307.0 | fea2.3-pza02194.1 | 132.73-180.31 | 5.66 | 9.35 | -47.86 | 16.08 | 0.34 | PD | CML441 |
|  |  | 5 | 367.0 | pza02207.1-pza01304.1 | 49.20-178.58 | 3.11 | 2.13 | -18.23 | -29.23 | 1.60 | OD | CML441 |
|  |  | 9 | 69.0 | pzb01110.6-pza01096.1 | 24.02-133.45 | 2.70 | 3.14 | -2.02 | -44.77 | 22.19 | OD | CML441 |
|  |  | 10 | 147.0 | pza01456.2-phm3844.14 | 135.93-146.55 | 2.61 | 4.24 | -22.06 | 42.00 | 1.90 | OD | CML441 |
|  | WS | 1 | 14.0 | phm14475.7-phm174.13 | 256.24-294.94 | 3.41 | 3.34 | -1.45 | 23.64 | 16.28 | OD | CML441 |
|  |  | 1 | 487.0 | pza03183.5-pza03189.4 | 46.06-64.26- | 3.48 | 4.24 | -3.63 | -23.44 | 6.45 | OD | CML441 |
|  |  | 2 | 300.0 | pza01885.2-pza02418.2 | 206.88-214.64 | 3.81 | 4.83 | -17.29 | 3.62 | 0.21 | A | CML441 |
|  |  | 3 | 226.0 | pza00279.2-pza02616.1 | 52.80-210.16 | 10.02 | 15.71 | 26.65 | 22.90 | 0.86 | PD | CML444 |
|  |  | 5 | 120.0 | pza03167.5-pza03339.2 | 207.60-210.89 | 3.87 | 7.28 | -1.60 | -32.45 | 20.31 | OD | CML441 |
| CYL | WW | 1 | 47.0 | pzb01227.6-pza00623.3 | 288.44-293.63 | 3.54 | 4.35 | 20.96 | -39.32 | 1.88 | OD | CML444 |
|  |  | 1 | 567.0 | phm595.30-pza02087.2 | 281.81-284.05 | 2.63 | 2.46 | 23.29 | -16.93 | 0.73 | PD | CML444 |
|  |  | 4 | 308.0 | fea2.3-pza02194.1 | 132.73-180.30 | 8.18 | 13.70 | -60.33 | -5.41 | 0.09 | A | CML441 |
|  |  | 5 | 368.0 | pza02207.1-pza01304.1 | 49.20-178.58 | 3.00 | 1.82 | -3.27 | -35.11 | 10.75 | OD | CML441 |
|  |  | 10 | 105.0 | pza01001.2-phm3736.11 | 146.54-147.76 | 2.70 | 3.53 | -30.97 | 15.36 | 0.50 | PD | CML441 |
|  |  | 10 | 143.0 | pza01456.2-phm3844.14 | 135.93-146.55 | 2.54 | 3.29 | -27.47 | 20.20 | 0.74 | PD | CML441 |
|  | WS | 1 | 44.0 | pzb01227.6-pza00623.3 | 288.44-293.63 | 2.57 | 1.25 | 9.25 | 6.53 | 0.71 | PD | CML444 |
|  |  | 1 | 487.0 | pza03183.5-pza03189.4 | 46.06-64.26 | 2.73 | 1.22 | 9.92 | -11.29 | 1.14 | D | CML444 |
|  |  | 2 | 135.0 | phm482.27-pza02727.1 | 11.10-227.92 | 3.78 | 3.73 | 16.02 | -25.03 | 1.56 | OD | CML444 |
|  |  | 2 | 236.0 | pza00365.2-pza02337.4 | 1.22-15.50 | 7.35 | 7.58 | -26.14 | -15.49 | 0.59 | PD | CML441 |
|  |  | 2 | 314.0 | pza02418.2-pza01352.5 | 214.64-226.45 | 2.64 | 2.02 | -12.54 | 2.65 | 0.21 | A | CML441 |
|  |  | 3 | 226.0 | pza00279.2-pza02616.1 | 52.80-210.16 | 16.08 | 20.71 | 29.55 | 35.10 | 1.19 | D | CML444 |
|  |  | 4 | 127.0 | phm2438.28-pza01905.12 | 3.55-244.08 | 4.31 | 3.42 | 15.11 | -23.23 | 1.54 | OD | CML444 |
|  |  | 5 | 167.0 | pza02015.11-pza03339.2 | 207.27-207.43 | 3.84 | 3.68 | 19.39 | 0.64 | 0.03 | A | CML444 |
|  |  | 7 | 68.0 | pza01909.2-pza01210.1 | 6.43-75.09 | 3.73 | 2.69 | -16.93 | 3.41 | 0.20 | A | CML441 |
|  |  | 8 | 158.0 | pza01210.1-pza01691.1 | 5.99-11.62 | 3.97 | 4.64 | 14.25 | -22.92 | 1.61 | OD | CML444 |
|  |  | 10 | 97.0 | pza01001.2-phm3736.11 | 146.53-147.76 | 2.71 | 2.39 | -5.97 | -20.14 | 3.37 | OD | CML441 |
| EH | WW | 1 | 333.0 | pza03200.2-pza02741.1 | 148.69-161.07 | 5.57 | 8.42 | 3.47 | -0.07 | 0.02 | A | CML444 |
|  |  | 4 | 4.0 | pza01477.3-pza01187.1 | 172.30-177.67 | 6.21 | 9.86 | 3.37 | -3.33 | 0.99 | D | CML444 |
|  |  | 10 | 270.0 | phm5740.9-pzb01301.5 | 8.77-9.75 | 4.42 | 6.88 | -2.04 | 2.96 | 1.46 | OD | CML441 |
|  |  | 10 | 316.0 | phm15868.56-pza02527.2 | 137.13-148.49 | 2.64 | 2.43 | -1.78 | -2.11 | 1.19 | D | CML441 |
|  | WS | 1 | 310.0 | pza02467.10-phm5622.21 | 183.83-196.92 | 6.52 | 8.39 | 3.10 | -0.61 | 0.20 | A | CML444 |
|  |  | 2 | 25.0 | phm6111.5-pza01374.1 | 21.99-28.31 | 3.93 | 4.30 | 2.14 | 0.93 | 0.43 | PD | CML444 |
|  |  | 3 | 212.0 | phm5502.31-pza00210.9 | 29.69-67.28 | 3.83 | 4.09 | 2.09 | -0.08 | 0.04 | A | CML444 |
|  |  | 4 | 98.0 | pza00529.4-pza03322.5 | 240.76-242.02 | 6.15 | 6.77 | 2.18 | 1.25 | 0.58 | PD | CML444 |
|  |  | 5 | 127.0 | pza03167.5-pza03339.2 | 207.60-210.89 | 2.74 | 2.83 | 1.46 | 1.22 | 0.84 | PD | CML444 |
|  |  | 7 | 62.0 | pza01909.2-pza01210.1 | 6.43-75.09 | 5.26 | 7.13 | 2.29 | 1.33 | 0.58 | PD | CML444 |
|  |  | 10 | 208.0 | pza02320.1-pza02398.2 | 99.47-132.25 | 3.89 | 8.01 | -3.67 | -0.54 | 0.15 | A | CML441 |
| PH | WW | 2 | 251.0 | pza02337.4-pza02450.1 | 15.51-47.58 | 4.76 | 7.03 | 3.45 | -0.16 | 0.05 | A | CML444 |
|  |  | 2 | 276.0 | pza03692.1-pza02890.4 | 166.65-187.22 | 2.90 | 3.98 | -0.84 | 3.30 | 3.93 | OD | CML441 |
|  |  | 3 | 42.0 | pza01447.1-pza00363.7 | 53.55-132.19 | 2.81 | 4.13 | 3.13 | 0.12 | 0.04 | A | CML444 |
|  |  | 4 | 81.0 | pza02779.1-phm5599.20 | 207.11-239.23 | 4.21 | 6.31 | 2.98 | 1.24 | 0.42 | PD | CML444 |
|  |  | 6 | 223.0 | pza02478.7-phm5529.4 | 141.11-167.12 | 3.17 | 11.95 | -3.12 | 5.28 | 1.69 | OD | CML441 |
|  | WS | 1 | 48.0 | pza00623.3-pzb01227.6 | 288.44-293.63 | 2.81 | 2.23 | 1.03 | 1.49 | 1.44 | OD | CML444 |
|  |  | 1 | 367.0 | csu1138.4-phm12323.17 | 53.35-119.01 | 5.92 | 7.70 | 2.74 | 0.89 | 0.33 | PD | CML444 |
|  |  | 2 | 177.0 | pza02264.5-phm13440.13 | 2.52-3.16 | 4.97 | 5.80 | 2.37 | 0.09 | 0.04 | A | CML444 |
|  |  | 2 | 350.0 | pza01352.5-pza02170.1 | 226.45-231.19 | 3.41 | 2.40 | 1.46 | -0.14 | 0.10 | A | CML444 |
|  |  | 3 | 210.0 | phm2343.25-pza02255.2 | 27.98-33.22 | 5.10 | 5.97 | 2.50 | -0.50 | 0.20 | A | CML444 |
|  |  | 4 | 273.0 | pza03459.1-pza02027.1 | 134.29-132.97 | 5.41 | 9.92 | -0.36 | -4.74 | 13.20 | OD | CML441 |
|  |  | 5 | 256.0 | ae1.7-pza00881.1 | 97.98-167.87 | 7.11 | 10.98 | -0.36 | 4.76 | 13.08 | OD | CML441 |
|  |  | 5 | 308.0 | pza03340.2-pza00222.7 | 20.20-58.57 | 4.37 | 5.92 | -0.81 | -2.96 | 3.67 | OD | CML441 |
|  |  | 7 | 68.0 | pza01909.2-pza01210.1 | 6.43-75.10 | 5.89 | 7.89 | 2.76 | 0.26 | 0.09 | A | CML444 |
|  |  | 9 | 71.0 | pzb01110.6-pza01096.1 | 24.03-133.45 | 2.63 | 1.72 | -1.30 | 0.23 | 0.18 | A | CML441 |

^1^Physical position of the marker flanking the QTL expressed in Mb (10^6^ pb). ^2^Genetic effects of the QTL are determined by the A: additives and D: dominant effects. QTL with additive effects are shown with positive values and were contributed by the parent CML444, and QTL with negative values are from the parent CML441. ^3^Gene action determined on the basis of the level of dominance was calculated using the ratio between dominant and additives effects of the QTL (|d/a|) according to Stuber et al. (1987) criterion: additive (A) = 0 – 0.20; partial dominance (PD) = 0.21 – 0.80; dominance (D) = 0.81 – 1.20, and overdominance OD > 1.20.


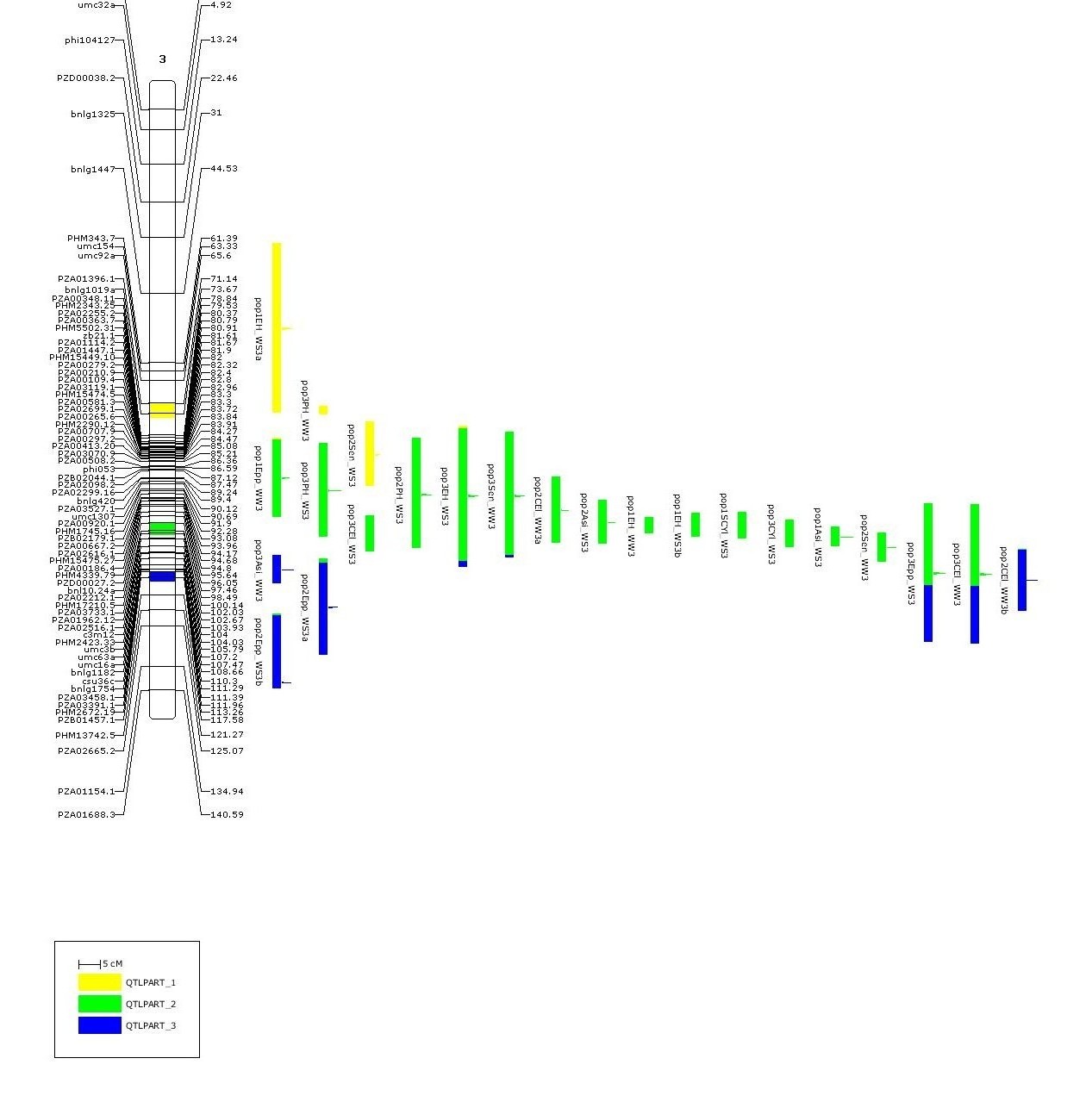
Figure S1. The meta-QTL analysis revealed a hotspot genomic region on chromosome 3 (green area) for morphophysiological traits related to drought tolerance in maize.
